# Development of a consumer involvement strategy for a small university‐based musculoskeletal research centre

**DOI:** 10.1002/msc.1806

**Published:** 2023-08-18

**Authors:** Rachel K. Nelligan, Travis Haber, Kim L. Bennell, Rana S. Hinman, Neil Bidgood, Jennifer Marlow, Belinda J. Lawford

**Affiliations:** ^1^ Centre for Health, Exercise and Sports Medicine Department of Physiotherapy School of Health Sciences The University of Melbourne Melbourne Victoria Australia; ^2^ Community‐based consumer representative, person with osteoarthritis Melbourne Victoria Australia

**Keywords:** consumer engagement, consumer involvement, musculoskeletal research, patient and community involvement

## Abstract

**Objective:**

To develop a Consumer Involvement Strategy which adheres to best practice recommendations and is feasible to implement in a small musculoskeletal research centre funded solely by external grants.

**Methods:**

The Strategy development involved five collaborative and iterative stages: (1) conceptualisation and initial consultation; (2) formation of the Consumer Involvement Strategy Action Group; (3) defining the scope and developing the strategy; (4) consultation and refinement; and (5) presentation and implementation. The final three stages were overseen by a Consumer Involvement Strategy Action Group comprising two post‐doctoral research fellows, a PhD student representative, and two consumers (people with osteoarthritis), all with experience in consumer involvement activities in research.

**Results:**

The final strategy aligns with best practice recommendations and includes five unique levels of consumer involvement that were devised to encompass the wide variety of consumer involvement activities across the research centre. It includes a policy document with five strategic aims, each supported by an implementation plan, and includes a suite of resources for researchers and consumers to support its application.

**Conclusion:**

The Consumer Involvement Strategy and its described development may serve as a template for other research teams facing similar resource constraints, both at a national and international level.

## 1 BACKGROUND

The inclusion of consumers and community members in health and medical research is now widely recognised as essential and expected internationally by funding bodies, government agencies, and the public (MACH, 2021; MRFF, 2023; NMHRC, 2016). This is due to the many benefits that consumer involvement can bring to the research process, and most importantly, the recognition that it is a fundamental right of consumers to participate in decision‐making that impacts them rather than being viewed solely as research subjects.

Various organisations have played a crucial role in integrating consumer involvement, also known as public involvement, in health and medical research on a global scale. For instance, INVOLVE, an initiative funded by the government of the United Kingdom (now succeeded by the National Institute for Health and Care Research (NIHR) Centre for Engagement and Dissemination), has significantly influenced consumer (public) involvement practices within the National Health Service. Similarly, the Patient‐Centered Outcomes Research Institute, a not‐for‐profit funding organisation, and the Canadian Institutes of Health Research, a federal health research funding agency, have played pivotal roles in advocating for the inclusion of consumers in health and medical research in the United States and Canada, respectively. In Australia, the National Health and Medical Research Council (NHMRC) has taken steps to facilitate the active participation of consumers and community members in all aspects of Australian health and medical research. In 2016, the NHMRC released a ‘Statement on Consumer and Community Involvement (CCI) in Health and Medical Research’ (NMHRC, 2016), and in 2020, it published a set of supporting resources called ‘The Toolkit’ (NHMRC, 2020). These resources serve as a guide for research organisations throughout Australia in implementing the NHMRC Statement.

To effectively and feasibly embed consumer involvement within a research organisation, it is recommended to co‐design context‐specific frameworks (Greenhalgh et al., 2019). Such tailored approaches are proposed to foster an organisation's culture and values surrounding consumer involvement and discourage superficial or checkbox approaches. Furthermore, tailoring can ensure the consumer involvement approach aligns with the researcher/organisation's available resources and funding. This is particularly important as resource constraints (e.g., time and cost) are a key researcher barrier to embedding consumer involvement in research (Ayton et al., 2022). In Australia, several consumer and professional organisations have developed practical tools for health and medical researchers to facilitate consumer involvement in the research process (Symons et al., 2023; WAHTN, 2021) and while research groups are likely to have structures in place, only a small number have taken the initiative to publish how they have operationalised the recommendations put forth by the NHMRC (Gunatillake et al., 2020; MACH, 2021; Miller et al., 2017). This includes the South Australian Health and Medical Research Institute (SAHMRI) (Miller et al., 2017; SAHMRI, 2020) and the Walter and Eliza Hall Institute (MACH, 2021), both large‐scale health and medical research Institutes, and OPUS, a research consortium and NHMRC Centre for Research Excellence in Total Joint Replacement (Gunatillake et al., 2020). Beyond this, we were unable to identify other examples to guide the development of a consumer involvement programme suitable for small research teams that rely exclusively on external competitive research funding.

The Centre for Health Exercise and Sports Medicine (CHESM) is a not‐for‐profit, multidisciplinary University‐based research centre made up of 37 researchers (including 10 PhD students) whose budget is entirely through external research grant funding. CHESM's research focuses on non‐surgical non‐pharmacological treatment strategies, particularly exercise for musculoskeletal conditions, to promote overall health and well‐being, with a key focus on osteoarthritis. Although CHESM has involved consumers in its research for over a decade, this has typically been ad hoc and primarily in a consultative role such as providing feedback on ethics documents, grant applications, intervention design, or pilot testing surveys. The Centre for Health Exercise and Sports Medicine now aims to expand and standardise its approach to consumer involvement and ensure future research is relevant to community needs.

This paper describes the development of CHESM's Consumer Involvement Strategy, which adheres to best practice recommendations and is feasible to implement long‐term for a small research centre whose budget is entirely through external research grant funding. Our approach may serve as a useful template for other researchers and research centres, both nationally and internationally.

## 2 METHODS

The development process was collaborative and iterative consisting of five key stages. The final three stages were overseen by a Consumer Involvement Strategy Action Group, which was established during stage two.

### 2.1 Stage 1. Conceptualisation and initial consultation

Consumer involvement was identified as a focus area during CHESM's 2022 research retreat, which brought together 10 research staff and six higher research degree students. A live and anonymous survey (via Poll Everywhere) was conducted to assess staff and students' perspectives of CHESM's existing approach to consumer and community involvement in research. Results showed varying levels of satisfaction (see Supporting Information S1: Appendix 1). In summary, half (*n* = 8) reported a lack of confidence with involving consumers in research activities, and few (*n* = 2, 13%) believed they had adequate training to do so. Furthermore, the majority (*n* = 15, 96%) believed it would be useful if CHESM had a specific strategy for consumer involvement in research. This was followed by discussions on how CHESM could increase consumer involvement in accordance with the NHMRC's Statement on Consumer and Community Involvement in Health and Medical Research (NMHRC, 2016).

Following the retreat, a team meeting was held to discuss further the perceived need for a CHESM‐specific Consumer Involvement Strategy. Before the meeting, a short anonymous online survey was completed by 17 staff and students. The aim of the survey was to assess staff/students' perceived level of importance related to involving consumers in CHESM research activities (see Supporting Information S1: Appendix 2). The survey is recommended by Western Australian Health Translation Network's Consumer and Community Involvement Program as a starting point to assess researcher commitment to consumer involvement and generate discussions in this area (WAHTN, 2021). The survey revealed that all respondents believed that consumer involvement in CHESM research is important, very important or critical to ensure relevance to community needs, to generate knowledge to improve healthcare, and satisfy the requirements of research funders. After further discussions, there was a consensus among staff and students to develop and implement a standardised strategy for consumer involvement within CHESM and to form a Consumer Involvement Strategy Action Group to oversee its development.

### 2.2 Stage 2. Formation of the Consumer Involvement Strategy Action Group

A committee with diverse skills and experiences was formed to lead the development of the strategy. The group consisted of two post‐doctoral research fellows (RKN [chair] and BJL), a PhD student representative (TH), and two consumers (JM: a person with hip osteoarthritis, NB: a person with knee osteoarthritis), all with experience in consumer involvement activities in research. The two consumer members had previously participated in a broad range of CHESM research activities including as a study participant, associate investigators on grant applications, members of working groups to inform development/test research interventions, and review of study finding lay summaries. The consumer members were invited to join the Action Group via email and phone and in appreciation of their participation, were offered a $250 gift voucher. The other members (staff and student representative) volunteered to be part of the Action Group.

### 2.3 Stage 3. Defining the scope and developing the strategy

CHESM's research activities span a wide range of consumer stakeholders, such as individuals with musculoskeletal conditions like osteoarthritis, health consumer advocacy groups, healthcare professionals, and peak professional bodies. However, for this strategy, the scope was confined to one stakeholder group, consumers—defined as people with lived experience of (or at risk of) musculoskeletal conditions and/or their support network (e.g., family, friends, and carers). This decision was made for practical reasons, with the aim of expanding the scope to include other stakeholders, such as health professionals, in the future.

The Action Group first conducted a search (via PubMed and Google) to identify existing consumer involvement frameworks that might be suitable to use as a guide. Several frameworks were identified based on their rigorous development (i.e., evidence‐based approach which included consumer involvement), transparent reporting, and their application to Australian research organisations. A summary of selected relevant frameworks is provided in Table 1. The selected frameworks were then reviewed by the Action Group and used to inform CHESM's own strategy for consumer involvement which was developed to address four organisational dimensions identified to contribute to the success of consumer involvement in research: governance, infrastructure, capacity, and advocacy (see Table 2) (Saunders & Girgis, 2011).

**TABLE 1:** Summary of relevant identified consumer involvement frameworks/documents.

Organisation	Framework/strategy title	Year finalised	Development methods	Brief description
Australian Health Research Alliance (AHRA) and Western Australian Health Translation Network (WAHTN)	Involving Consumers in Health and Medical Research: Consumer and Community Involvement (CCI) Handbook (WAHTN, 2021)	2021	An AHRA national initiative funded by the Medical Research Future Fund (MRFF)	Provides practical guidance for organisations, researchers, consumers, and funders on how to successfully embed consumer involvement in research
			Development led by WAHTN with input from their 23 state‐wide member partners (universities, medical research institutes, public and private hospitals, PathWest and the Department of Health)	
South Australian Health and Medical Research Institute (SAHMRI)	SAHMRI Consumer and Community Engagement Framework (Miller et al., 2017; SAHMRI, 2020)	2020	Overseen by a Steering Committee made up of senior representatives from SAHMRI and Health Consumers Alliance of South Australia	Outlines SAHMRI's consumer and community engagement processes across the organisation and all phases and stages of research
			Informed by a literature search and review; in‐depth, semi‐structured interviews with key internal (SAHMRI) and external stakeholders (including consumers and carers); and a consensus workshop with the SAHMRI Research Executive, consumers and researchers	
Australian Clinical Trials Alliance (ACTA)	ACTA Consumer Involvement and Engagement Toolkit (ACTA, 2021; Symons et al., 2023)	2019	A joint initiative between ACTA and Clinical Trials: Impact & Quality (CT:IQ). Developed by a working group of end‐users (researchers, research organisation representatives, and consumers) who ratified content and endorsed the definitions and acronyms used throughout	Provides practical advice for researchers and research organisations wishing to conduct patient‐centred clinical trials
Cancer Australia	National Framework for Consumer Involvement in Cancer Control (Cancer Australia, 2011)	2011	Overseen by a Project Steering Group. Developed via multiple methods including a 1‐year consultation phase comprising interviews and a Delphi process, a literature review and a national workshop. Consultation was with consumers, consumer organisations, health professionals, policy makers, researchers, and service planners from government, non‐government and professional organisations	A national framework designed to facilitate consistent approaches to consumer engagement in cancer control
				Offers principles to govern consumer engagement and guidance which can be adapted to local circumstances
The Centre for Research Excellence for OPtimising oUtcomes, equity, cost effectiveness, and patient Selection (OPUS) in Total Joint Replacement (OPUS)	OPUS Consumer and Community Involvement Programme (CCIP) (Gunatillake et al., 2020)	2020	Not described	A four‐tiered model where consumers nominate their level of involvement based on their interest/commitment level. The model aims to encourage consumers, in partnership with researchers, to improve the quality and integrity of orthopaedic research

**TABLE 2:** Description of the four organisational dimensions identified to contribute to the success of consumer involvement (Saunders & Girgis, 2011).

Dimension	Description
Governance	Refers to having appropriate structures within the research organisation to facilitate the engagement of consumers in research, as well as policies that encourage and support researchers to engage consumers in their research
Infrastructure	Refers to a range of tools and resources to support consumer involvement, such as registers of people interested in contributing to research, quality information materials that explain consumers' roles in the organisation, and how their participation is supported
Capacity	Refers to activities/training that aims to enhance the skills and capacity of both researchers and consumers
Advocacy	Refers to having advocacy and agenda‐setting from organisational leaders to ensure consumer involvement frameworks are implemented effectively across research organisations

During this development stage, unique ‘levels’ of consumer involvement were devised to encompass the wide variety of consumer involvement activities within CHESM (adapting the approach used by Gunatillake et al. (2020)). Consumer Involvement Levels were developed by identifying and expanding on the variety of ways consumers were currently involved in CHESM research activities and then by applying the research‐modified International Association for Public Participation spectrum (Inform; Consult; Involve; Collaborate; Empower) (Bammer, 2019). Onboarding, training, honorarium, and evaluation processes relevant to each level were then developed by the Action Group.

During this stage, the Action Group also liaised (via Zoom meetings and emails) with research organisations with well‐established processes for consumer involvement in research, such as the Walter and Eliza Hall Institute of Medical Research, Australia and the Melbourne Social Equity Institute, Australia. The main objective of these interactions was to seek feedback on the Action Group's developing approach and to establish connections for accessing valuable researcher resources related to consumer involvement.

### 2.4 Stage 4. Consultation and refinement

Following development, the draft strategy framework was presented in a meeting to the CHESM Advisory Board (eight members including the Head of Department, CHESM Director, a faculty representative, two clinician representatives, a stakeholder representative, and a consumer representative) for feedback. Their suggestions were incorporated into the final draft. Next, all 37 CHESM staff and students, and six consumers with prior experience in CHESM consumer involvement activities (in addition to those on the Action Group) were invited via email and phone to review and offer feedback on the strategy and its accompanying documents. Consumers were offered a $50 gift voucher in reimbursement for reviewing the documents. People were given the option to provide their feedback through various formats, such as comments and track changes within the documents, feedback summaries via email, and feedback provided via phone calls. The consultation/feedback period lasted a month (May 2023). Thirteen CHESM researchers (nine staff and four PhD students) and four people with osteoarthritis provided feedback. Feedback received was compiled and presented at a CHESM staff/student meeting, where additional feedback was collected. All feedback was then used to refine the documents and produce the final versions.

### 2.5 Stage 5. Presentation and implementation

Once the strategy was finalised, it was uploaded to a central online repository and presented to all centre staff and students in a 1‐h meeting. The presentation covered an overview of the strategy, as well as an orientation to all associated documents. An implementation plan was devised including plans to promote the strategy and its development via our social media channels and at relevant conferences to contribute ideas/examples of best practices to other research teams.

### 2.6 Patient and public involvement statement

Two people with osteoarthritis (NB and JM) are part of the Consumer Involvement Strategy Action Group, who developed this work and will oversee its ongoing implementation. Four additional people with osteoarthritis reviewed the draft Consumer Involvement Strategy and provided feedback (via email and telephone) during the 1‐month consultation period.

## 3 RESULTS

**FIGURE 1:**
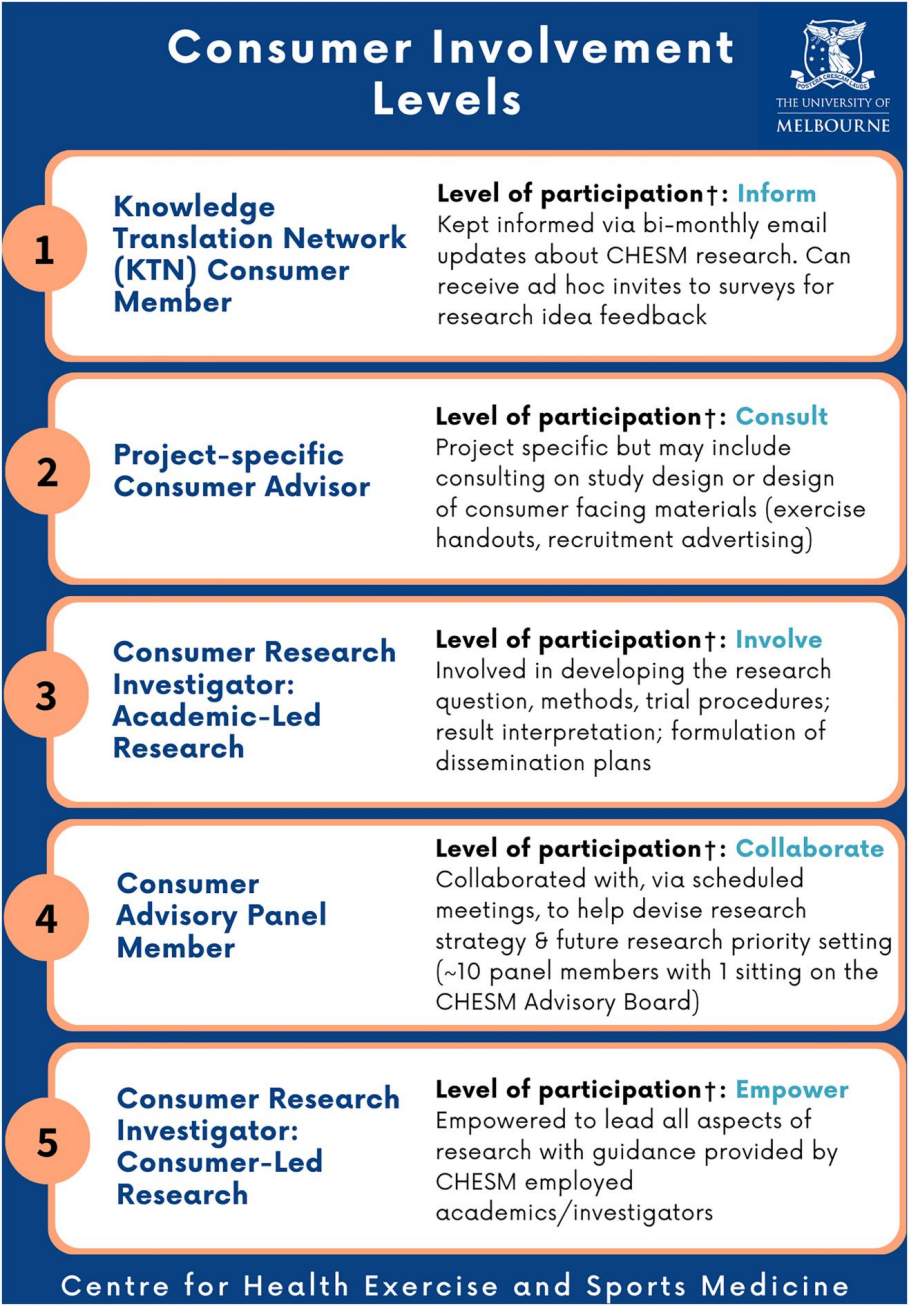
Overview of CHESM Consumer Involvement Levels. Consumer Involvement Levels and types of involvement are distinct from involvement as a study participant. A consumer may participate in multiple consumer activity levels at one time. For example, they may be a Knowledge Translation Network Consumer Member (level 1) and participate in a project specific activity as a Project‐specific Consumer Advisor (level 2). ^†^Level of participation as per the research‐modified IAP2 spectrum (Bammer, 2019). CHESM, Centre for Health Exercise and Sports Medicine; IAP2, International Association for Public Participation.

The CHESM Consumer Involvement Strategy was implemented July 2023. All Strategy documents and resources have been made publicly available and up‐to‐date versions can be accessed via the CHESM webpage (see https://healthsciences.unimelb.edu.au/departments/physiotherapy/chesm#involvement). The strategy applies to the five levels of CHESM consumer involvement activities devised. An overview of each level is presented in Figure 1, and the associated activities, onboarding, training, honorarium, and evaluation processes for each level can be found on the CHESM webpage. The strategy is described below according to the four dimensions of governance, infrastructure, capacity, and advocacy.

### 3.1 Governance

CHESM's Consumer Involvement Strategy is overseen by the Consumer Involvement Action Group and CHESM's Director (KLB) and Deputy Director (RSH). At its core is a Policy document (the CHESM Consumer Involvement Strategy Policy Document), which was adapted from the Australian Clinical Trials Alliance Framework for Consumer Involvement Strategy v1 (ACTA, 2021). The Policy document (see CHESM webpage) details CHESM's commitment to consumer involvement and includes five strategic aims: (i) promoting inclusivity and establishing a supportive and safe culture, (ii) building capacity, (iii) building capability, (iv) continuously improving, and (v) collaborating, networking, and disseminating. An implementation plan with milestones and timeframes is provided for each strategic aim, and an annual policy review is scheduled to assess progress and update the policy milestones to ensure continued implementation and governance.

To further ensure effective governance and implementation of the strategy and its policy, CHESM has made consumer involvement a regular agenda item in its monthly team meetings. This provides a forum for discussing ongoing and planned consumer involvement activities across CHESM and allows researchers to raise any challenges faced when implementing the strategy. In addition, all CHESM clinical trial protocols will include a consumer and community involvement statement and the reporting of clinical trials will adhere to the Guidance for Reporting Involvement of Patients and the Public (GRIPP2‐SF) (Staniszewska et al., 2017). This checklist is a universally accepted guideline to improve reporting of consumer involvement in research.

### 3.2 Infrastructure

To support CHESM's Consumer Involvement Strategy, a range of infrastructure was developed (in addition to the Policy document), including a suite of resources for CHESM staff/students to use to implement the strategy. These documents are summarised in Table 3 and are available to view on the CHESM webpage.

**TABLE 3:** Summary of documents developed to support the implementation of CHESM's Consumer Involvement Strategy.

Document end‐user	Document name	Description
CHESM staff/student	Honorarium schedule	Wherever permitted by the external funding body, costings for consumer involvement activities are to be completed as per CHESM's Honorarium schedule
	Consumer involvement checklist for levels 2, 3 and 5	A checklist for staff/students to use to help apply the strategy. The checklist forms part of staff/student training
Consumer	Consumer role description	Template adapted from ACTAs Toolkit^a^
		To be tailored by lead researchers and provided for consumer involvement activities within levels 2–5^b^. Forms part of CHESM's Consumer Representative Support Resources
	Conflict of interest form	Conflict of Interest declaration to be provided to consumers in involvement levels 2–5^b^
	Out‐of‐pocket reimbursement form	Template adapted from ACTAs Toolkit^a^
		To be used to claim reimbursement for out‐of‐pocket parking expenses related to consumer involvement activities. To be provided to consumers in involvement levels 2–5^b^. Forms part of CHESM's Consumer Representative Support Resources
	Consumer thank you letter	To be provided to consumers in involvement levels 2–5^b^ on completion of involvement
	Consumer plain language statement review guide	Template adapted from ACTAs Toolkit^a^
		Provides instructions to assist consumers review study lay summaries. To be provided to consumers in involvement levels 2–5^b^, when relevant. Forms part of CHESM's Consumer Representative Support Resources
	Consumer focus group ground rules	Template adapted from ACTAs Toolkit^a^
		Provides ground rules for meeting/focus group participation. To be provided to consumers in involvement levels 2–5^b^, when relevant. Forms part of the Consumer Representative Support Resources

Abbreviations: ACTA, Australian Clinical Trials Alliance; CHESM, Centre for Health Exercise and Sports Medicine; KTN, Knowledge Translation Network.

^a^ References: (ACTA, 2021; Symons et al., 2023).

^b^ CHESM Consumer Involvement Levels: 1: KTN Consumer Member; Level 2: Project‐Specific Consumer Advisor; Level 3: Consumer Research Investigator: Academic‐Led Research; Level 4: Consumer Advisory Panel Member; Level 5: Consumer Research Investigator—Consumer‐Led Research.

In addition, Consumer Involvement Process Evaluation Surveys were developed (a researcher version and a consumer version, see Supporting Information S1: Appendices 3 and 4) to be used to evaluate CHESM's consumer involvement in research and inform process refinements. These were adapted by the Action Group from the example provided in ACTA's Consumer Involvement and Engagement Toolkit (ACTA, 2021) and include a mix of questions with Likert scale and open text response options. The consumer version of the survey was developed to gather feedback from consumers regarding their experience and perspectives on their involvement in activities/a project. It asks questions about their understanding of their role, including what they could and could not influence and their perceptions regarding the recognition and value placed on their contributions, the adequacy of training and support they received, the acquisition of new skills and knowledge, the support provided by CHESM, the sufficiency of time provided for feedback, the extent of project updates received, and the fairness of honorarium/reimbursement. The researcher version of the survey was developed to gather feedback on the researchers' experience and perspectives regarding consumer involvement. It asks questions about the influence of consumer involvement on project design, the perceived value and usefulness of consumer contributions, the timeliness of feedback from consumers, the adequacy of training and support received to effectively involve consumers, whether the researchers considered consumer involvement to be a good use of project resources, and the ease of the overall process. It is recommended that process evaluations be completed (within REDCap^TM^ software (Harris et al., 2009; Harris et al., 2019)) independently by consumers and the lead researcher for all activities associated with CHESM Consumer Involvement Levels 2–5 and be completed immediately after consumer involvement activity ends or 6 monthly if a consumer involvement activity is planned to span >1 year. Completed surveys are monitored by an appointed staff Consumer Involvement Representative, who reports findings at monthly CHESM team meetings and highlights areas for refinement/improvement in future consumer involvement activities.

A key part of CHESM's strategy is maintaining the already established CHESM Knowledge Translation Network, a registry of consumers (and other end‐users, including clinicians) interested in CHESM research activities. Consumer Knowledge Translation Network members comprise the Strategy's Consumer Involvement Level 1 (see Table 2). The Knowledge Translation Network was established prior to the development of this strategy and currently has 7381 consumer members and 4714 clinician members since its inception in 2020. Annual targets for Knowledge Translation Network consumer member registrations will be set, and monthly meetings will be held, to review progress and discuss marketing strategies to increase registrations and consumer interest throughout the year.

### 3.3 Capacity

To build researcher capacity, orientation to the Consumer Involvement Strategy and its associated resources (uploaded and accessible to all staff/students on the online CHESM research repository) was conducted at a 1‐h team meeting. Training also covered the values of consumer involvement. Additionally, a student Consumer Involvement Representative will be appointed each year (for a 1‐year term) to train new graduate research students in implementing the strategy. Similarly, a staff Consumer Involvement Representative will train new staff and review consumer involvement process evaluation surveys to identify training needs and areas for improvement, which will be presented at CHESM team meetings. As per the strategy's policy document, a range of staff/student educational sessions will be held every year with invited speakers covering key topics, including engaging and respectfully working with Aboriginal and Torres Strait Islander Peoples and culturally and linguistically diverse consumers, LGBTIQA+ inclusive practices and co‐design research methods.

To build the capacity of consumer representatives, consumers involved in activities associated with Consumer Involvement Levels 2–5 will be provided with a range of recommended resources (CHESM's Recommended Consumer Representative Support Resources). These are a range of resources modified/compiled from various sources, including ACTA's Consumer Involvement and Engagement Toolkit, WAHTN's CCI Handbook, and WAHTN's CCI Program.

### 3.4 Advocacy

CHESM's Director (KLB) has demonstrated a strong commitment to consumer involvement by providing support and funding to develop this Consumer Involvement Strategy. The Director's ongoing dedication to overseeing the strategy's implementation is further evidenced by their inclusion of consumer involvement as a regular agenda item in monthly team meetings and their participation in annual reviews of this strategy and its policy. This ongoing support and advocacy for consumer involvement ensures that it remains a top priority for CHESM in the long term.

## 4 DISCUSSION

CHESM's Consumer Involvement Strategy was developed to align with best practice recommendations for consumer involvement in health and medical research while being feasible to implement for a small research team whose budget is entirely from external competitive research grant funding. The strategy targets identified capability and opportunity facilitators of consumer involvement in health and medical research (Ayton et al., 2022). To build researcher capability, a variety of annual training activities will be delivered including strategy implementation processes, engaging underrepresented consumer groups, and adopting consumer‐centric research designs. To build consumer capability, a comprehensive collection of ‘Consumer Representative Support Resources’ has been compiled. To build opportunity, the strategy outlines clear systems and processes to facilitate researchers to involve consumers throughout the entire research cycle.

One of the key strengths of the strategy is the inclusion of individual process evaluation surveys to be completed by both researchers and consumers immediately following the conclusion of consumer involvement. These surveys will provide valuable insights regarding the satisfaction of consumers and researchers with the consumer involvement process, allow identification of areas for improvement and future training needs, and will inform strategy refinements. A potential limitation of the strategy is the intentional omission of evaluating how consumer involvement activities impact research outcomes and outputs. From a research and funding organisation perspective, it is recognised as important to assess the research impacts/outcomes resulting from consumer involvement to demonstrate the value and return on investment of including consumers in the research process (Esmail et al., 2015). Potential indicators of the impact/outcomes of consumer involvement on research include funding opportunities, recruitment/retention rates, translation/dissemination of research results, and research relevance, as well as broader downstream impacts such as health outcomes, changes in health service utilisation, and cost savings (DeBortoli et al., 2022). Although a rapid review of existing tools identified four potential options to measure the impact/outcomes of consumer involvement on health research outcomes in Australia (AHRA, 2021), we found that these tools were not suitable for our specific context. We believed a single tool would not adequately capture the diverse range of consumer involvement activities conducted in our centre and that the length of these tools, which could take up to 40 min to complete, may impose a burden on both consumers and researchers. Furthermore, we did not feel confident that we could establish causality in relation to the impact of our consumer involvement on short or long‐term research outcomes/outputs. Another potential limitation is the current absence of explicit targets and strategies for reaching/involving underrepresented consumer groups (e.g., Aboriginal and Torres Strait Islander Peoples and culturally and linguistically diverse consumers). As CHESM has only just started routinely collecting gender, ethnicity, and socioeconomic participant data in our research studies and have not previously collected these data from our consumer representatives, we did not feel ready to set diversity targets. However, as outlined in the policy document of our strategy, we will ensure these data are routinely collected to inform future annual targets and, moving forward, we will work with delegates from underrepresented groups and strive to have consumer involvement representatives who span the diverse characteristics and backgrounds of our research audience.

## 5 CONCLUSION

This paper presents a Consumer Involvement Strategy that aligns with best practice recommendations and was specifically designed for a small University‐based research centre that is dependent on external research grant funding. The development process is clearly explained, and the finalised documents are made available to serve as a potential template for other research teams facing similar resource constraints, both at a national and international level.

## AUTHOR CONTRIBUTIONS

Rachel K. Nelligan, Kim L. Bennell, Rana S. Hinman and Belinda Lawford conceived the concept. Rachel K. Nelligan formed and chaired the Consumer Involvement Strategy Action Group. All author's contributed to the development of the Consumer Involvement Strategy and its associated documents, contributed to writing the manuscript and approved the final manuscript.

## CONFLICT OF INTEREST STATEMENT

None declared by all authors.

## ETHICS STATEMENT

Not applicable.

## Supporting information

Supporting Information S1

## Data Availability

Data sharing is not applicable to this article as no new data were created or analysed in this study.
