# Supplementary material for: Development of a consumer involvement strategy for a small university‐based musculoskeletal research centre
Source: Musculoskeletal Care. 2023 Aug 18;21(4):1238–47. doi: 10.1002/msc.1806 (PMC10947031; doi:10.1002/msc.1806)
Supplement: Supplementary file 1 — Supporting Information S1 [file MSC-21-1238-s001.pdf]

Appendix 1: Results from an anonymous survey (via Poll Everywhere) assessing staff and student's perspectives of CHESM's existing approach to consumer and community involvement in research

Question	N	%
1. How would you rate your level of awareness of CHESM's overall approach to public and patient engagement?		
High level of awareness	8	50%
Some level of awareness	8	50%
Neither aware nor unaware	0	0%
Low level of awareness	0	0%
Completely unaware	0	0%
Total	16	100%
2. At what stage would you say CHESM is when it comes to routinely engaging the public and/or patients in its activities?		
Well established	0	0%
Established/making some progress	12	75%
Just beginning	4	25%
Not yet started	0	0%
Total	16	100%
3. Do you think you have adequate training in public and patient engagement?		
Strongly agree	0	0%
Agree	2	13%
Neither agree nor disagree	3	19%
Disagree	10	63%
Strongly disagree	1	6%
Total	16	100%
4. How confident do you feel in your ability to involve consumers in a research project?		
Very confident	0	0%
Reasonably confident	8	50%
Not very confident	8	50%
Not confident at all	0	0%
Total	16	100%
5. CHESM uses the input generated from PPE initiatives (eg to influence decisions, to change intervention designs etc)		
Strongly agree	0	0%
Agree	7	44%
Neither agree nor disagree	9	56%

Disagree	0	0%
Strongly disagree	0	0%
Total	16	100%

6.Overall, I believe CHESM has an appropriate level of public and patient engagement activity.		
Strongly agree	0	0%
Agree	5	31%
Neither agree nor disagree	3	19%
Disagree	8	50%
Strongly disagree	0	0%
Total	16	100%

7. It would be useful if CHESM had a specific strategy or framework to follow for public and patient engagement in our research projects.		
Strongly agree	15	94%
Agree	0	0%
Neither agree nor disagree	1	6%
Disagree	0	0%
Strongly disagree	0	0%
Total	16	100%

8. CHESM should use more formal co-design methods to involve consumers in our trials?		
Strongly agree	6	38%
Agree	6	38%
Neither agree nor disagree	4	25%
Disagree	0	0%
Strongly disagree	0	0%
Total	16	100%

Appendix 2: Results from an anonymous survey assessing staff and student's perceived level of importance related to involving consumers in CHESM research activities (n=17). Survey taken from the Western Australian Health Translation Network's (WAHTN) Consumer and Community Involvement Program (CCIP).

Statements	Not important (n, %)	Desirable (n, %)	Important (n, %)	Very important (n, %)	Critical (n, %)
**1.** The insights and perspectives of consumers/patients are important for research	0 (0%)	0 (0%)	3 (18%)	10 (59%)	4 (24%)
**2. Consumer involvement is needed to:**					
Ensure that the research being conducted is relevant to community needs	0 (0%)	0 (0%)	1 (6%)	9 (53%)	7 (41%)
Ensure research generates knowledge that could improve healthcare, education or training	0 (0%)	0 (0%)	0 (0%)	13 (76%)	4 (24%)
Improve public awareness of, and support for, science and research	0 (0%)	3 (18%)	2 (12%)	8 (47%)	4 (24%)
Facilitate effective translation of research to deliver improved health outcomes	0 (0%)	1 (6%)	0 (0%)	9 (53%)	7 (41%)
Increase public confidence in research through enhanced openness and transparency in the conduct of research	0 (0%)	2 (12%)	3 (18%)	10 (59%)	2 (12%)
Increase public confidence in research through improved accountability over the use of public funds	0 (0%)	3 (18%)	3 (18%)	9 (53%)	2 (12%)
Help develop a greater understanding and awareness of research	0 (0%)	2 (12%)	4 (24%)	8 (47%)	3 (18%)
Satisfy requirements of research funders	0 (0%)	0 (0%)	3 (18%)	12 (71%)	2 (12%)
Meet ethics requirements	3 (18%)	2 (12%)	5 (29%)	7 (41%)	0 (0%)
Comply with policy requirements of research organisation	2 (12%)	2 (12%)	6 (35%)	7 (41%)	0 (0%)
Provide a different, non-scientific viewpoint	0 (0%)	1 (6%)	1 (6%)	8 (47%)	7 (41%)

Appendix 3: Researcher Process Evaluation Survey (Version 1)

This survey asks you to reflect on your experience working with consumer expert/s on a research study and what worked well, or maybe didn't work well.

Please enter the name of the project you are evaluating:

____

Which consumer involvement level were consumers part of? (Select all that apply)

- Level 2: Project-specific Consumer Advisor. (This role can involve a range of tasks including providing feedback on documents (e.g. study consent forms), helping design or test an intervention or treatment, or being part of a focus group discussion)
- Level 3: Consumer Research Investigator - Academic-Led Research (This role involves designing and conducting a research trial with CHESM researchers)
- Level 4: Consumer Advisory Panel Member. (This role involves being a Consumer Advisory Panel member and advising on CHESM strategy & future research priority setting and may include attending CHESM Advisory Board meetings 1-2 times a year)
- Level 5: Consumer Research Investigator- Consumer-Led research (This role involves leading a research trial with guidance/mentorship provided by CHESM researchers)
- I don't know

Briefly, what did the consumer's role in the project involve – what things did you ask them to do? (For example, what was in the 'Consumer Role Description'?)

____

What were your overall impressions about their involvement?

____

Please read each statement below and tell us how much you agree with each.

**I feel that…**	Strongly disagree	Disagree	Neither agree nor disagree	Agree	Strongly agree
	1	2	3	4	5
the consumer involvement influenced the project design					
the consumer(s) made valuable/useful contributions to the project					
when asked for feedback, consumers responded in a timely manner					
I had enough training/support to know how to involve consumers					

the consumer engagement was a good use of our project resources					
the process of involving consumers was easy					
the majority of suggestions raised by consumers was implemented					
the majority of suggestion or comments made by consumers were addressed or acknowledged irrespective of implementation					

If consumer suggestions were not implemented, why not?

____

What do you think is working well in how CHESM involves consumers in research activities?

____

What challenges have you faced in involving consumers in your research activities?

____

How could we improve our consumer involvement process/approach?

____

Do you have anything else to add?

____

Appendix 4: Consumer Process Evaluation Survey (Version 1)

Thank you for your involvement in our research at CHESM. We greatly value your contribution and commitment to
our research.

This survey asks you to reflect on your experience in our research as a consumer expert (not as a research participant).

We'd value your feedback and suggestions about how we could have improved your experience.

Please enter your name (optional):

____

Which CHESM consumer involvement level was your role?

- Level 2: Project-specific Consumer Advisor. (This role can involve a range of tasks including providing feedback on documents (e.g. study consent forms), helping design or test an intervention or treatment, or being part of a focus group discussion)
- Level 3: Consumer Research Investigator - Academic-Led Research (This role involves designing and conducting a research trial with CHESM researchers)
- Level 4: Consumer Advisory Panel Member. (This role involves being a Consumer Advisory Panel member and advising on CHESM strategy & future research priority setting and may include attending CHESM Advisory Board meetings 1-2 times a year)
- Level 5: Consumer Research Investigator- Consumer-Led research (This role involves leading a research trial with guidance/mentorship provided by CHESM researchers)
- I don't know

Please enter the project you have been involved in as a consumer expert. If you have been involved in more than one project, we ask that you please evaluate each separately. You can add another project at the end of this survey. If you would prefer not to provide details about the project you were involved in to remain anonymous, you can skip this question.

____

Briefly, what did your role in the project involve - what things have you been asked to do?

____

What were your overall impressions about your involvement?

____

Please read each statement below and tell us how much you agree with each.

**I feel that…**	Strongly disagree	Disagree	Neither agree nor disagree	Agree	Strongly agree
	1	2	3	4	5

I am clear about what my role is/was (including what I could and could not change about the project)					
I have made a contribution to the research/project					
My contribution has been valued					
I had satisfactory training/support to undertake my role					
I have gained new skills/knowledge that are useful (for example, I learnt more about the disease/condition being researched)					
I have been provided with satisfactory support from CHESM to do my role					
When asked for feedback, I have been given enough time to respond/provide feedback					
I have been kept up to date about the project					
I have been appropriately reimbursed for my time on the project					

What do you think is working well in how CHESM involves consumers in research activities?

____

What challenges have you faced in providing your input (as a consumer expert) into our research activities?

____

How could we have involved or supported you better when providing input into our research?

____

Do you have anything else to add?

____
